# Attractive but Harmful Commodities: Looking Across Unhealthy Commodities to Improve Public Health Responses

**DOI:** 10.1002/puh2.70117

**Published:** 2025-09-12

**Authors:** Robin Room

**Affiliations:** ^1^ Centre For Alcohol Policy Research La Trobe University Melbourne Australia; ^2^ Centre for Social Research on Alcohol and Drugs, Department of Public Health Sciences Stockholm University Stockholm Sweden

## Abstract

Public health discussions now apply the label of ‘unhealthy commodities’ to items attractive to consumers but which bring harm to health and welfare. The list includes tobacco, alcohol, cannabis and other drugs, sugar‐sweetened beverages, overprocessed foods and commercial gambling. The histories of markets and controls in such commodities vary, with commerce in alcohol, drugs and gambling prohibited in many places at times during the last two centuries, but few limits on sugar, or on tobacco until recent years. In the current world, regimes vary for retail sale between commodities and in different jurisdictions. Some examples are given of successful control measures which might be more widely applied: measures such as licensing of retail sales; closing off web‐based sales; and imposing minimum unit pricing. The new thinking in public health offers the opportunity to look across what have been separate fields of action and experience and to learn from case study examples of effective mechanisms to reduce rates of harmful consequences from unhealthy commodities.

## 1 Introduction

In recent years, public health discussions have applied the label of ‘unhealthy commodities’ to describe a diverse range of items which are attractive to consumers but bring harm to health and welfare (e.g., [1, 2, 3]). Such commodities are often attractive to consumers, and thus as items of commerce, but may cause considerable health and welfare harm—both to the users and to those around them. The list of unhealthy commodities includes psychoactive substances such as tobacco, alcohol and cannabis, along with other ‘drugs’, mostly internationally illicit. As used in the public health discussions, the list of ‘unhealthy commodities’ also includes commercial gambling (e.g., [1, 2, 3]) and attractive but potentially harmful foods—sugar‐sweetened beverages and what are characterised as ‘ultraprocessed foods’ [4, 5]. Nestle [6] has offered a ‘working definition’ of these: that they are ‘not obviously related to the whole foods from which they are derived’; that they are ‘formulated with industrially produced ingredient additives’ and ‘to be hyperpalatable’. She adds that they are ‘heavily marketed, attractively and conveniently packaged, relatively inexpensive [and] highly profitable.’

Public health‐oriented discussions are focused on means of limiting the purchasing and use of such unhealthy commodities. There is a growing history of efforts at market control on attractive but unhealthy commodities and at persuading consumers not to purchase and use them. But the efforts have tended to be focused on particular categories, and there has been relatively little effort to look across the different categories and to think about how a measure which has worked for one commodity category might be applied to another. Thinking about how a measure which has worked in one jurisdiction might be applied in another has also been somewhat limited. This article considers some exemplary cases where it seems that a measure which has worked in one place or for one commodity might well be further applied to another or in another place, to provide examples which may encourage some more thinking across commodities and jurisdictions.

## 2 The State and Unhealthy Commodities: A Brief History

As discussed below, there are examples reaching back centuries of policies limiting or forbidding commerce in some of the unhealthy commodities. But European empires long used such commodities as a fiscal resource—historical discussions have described some of the commodities as having been in the past the ‘glue of empires’ [7, 8], providing financing and binding their parts together. For instance, tobacco, alcohol and sugar were major commodities for European empire‐building in the West Indies, with products exported to the ‘home market’ and elsewhere; and export of opium to China was a major fiscal support of the British and other European empires in India and elsewhere in Asia [7, 9, 10]. In the wake of the 19th century international temperance movements, such trade in at least alcohol and opium declined [11, 12]. Thus, alcohol use and trading were prohibited in the United States and a dozen other countries in the two decades after 1915 [11], and reducing the nonmedical opium trade was a major focus of the international drug control treaties initiated around that time [13].

Such thinking and limits were not applied in those days for sugar. In addition, most of the other food commodities considered ultraprocessed did not exist then. And, although commercial gambling was limited by law or forbidden in many countries in the 19th and early 20th centuries, it was ‘legalised and commercialised on grand scale only since the 1960s’ ([14], p. 1).

## 3 The Situation Nowadays

Public health and welfare responses to limit the harms have varied between products and across societies and time. Opium was freely available before the late 19th century, but as noted, nonmedical use became prohibited by international treaties after 1918. Tobacco was freely available until after 1950; now it is heavily taxed in Australia and increasingly taxed elsewhere, forbidden for children, and its advertising limited. An international Framework Convention on Tobacco Control, negotiated under World Health Organisation (WHO) auspices, calls for measures to limit its advertising and market availability, with some limited success [15]. As a market comestible, alcohol is in the jurisdiction of the Food and Agriculture Organisation, with international advice and controls under the 1994 General Agreement on Tariffs and Trade, but it is treated as a separate item from foodstuffs, with international advice on controls coming primarily from the WHO [16, 17]. Legal arrangements in high‐income countries primarily include licensing and regulation of alcohol sellers, excise taxes which raise its price, some limits on advertising and on sale hours and no sales to children [18]. Commercial gambling sales were legalised in many societies after 1945; usually, sellers are licensed, and there are restrictions of varying severity and implementation on advertising [19]. As discussed below, the advent of online gambling sales in the last quarter‐century has substantially limited control by governments over the gambling market.

Each product has a different regime of licensing for retail sale: generally stronger control regimes for opium and alcohol than for gambling or tobacco. For sugar products, there is no separate market control regime from other foodstuffs, but sugared beverages are more or less specifically taxed for about half the world population [20].

## 4 Frameworks for Control of Harmful Commodities

Although for ‘illicit drugs’ the policies and procedures have been coordinated under the international ‘narcotic drug’ treaties, regulation and enforcement for other unhealthy commodities have developed separately, and often quite differently in different jurisdictions. In recent years, the categories of control regimes have increased, with cannabis increasingly moving out of the drug treaty control system in several countries and jurisdictions, and with nonmedical use becoming legal in an increasing number of places [21, 22, 23].

It is in the public health interest to compare across commodities and jurisdictions the systems of licensing and control, allowing a discussion of how the systems might be better coordinated, with the control efforts proportional to the controlled product's harm. This article thus considers paths forward for practical application of the public health field's growing tendency to look across the control systems, considering ‘unhealthy commodity’ industries in a common frame. The question we are seeking to answer is as follows: Are there policies and regulations which have proved successful in controlling the market in one unhealthy commodity and jurisdiction which could be applied also to other unhealthy commodities and elsewhere?

Discussing the regulation of the food industry, Nestle [6] offers a listing of potential measures which governments can take: dietary guidelines; media advertising campaigns; warning labels; marketing restrictions; taxes to increase relative costs; portion‐size restrictions; and farm subsidies for healthier alternatives. Looking more broadly at unhealthy commodities, we might subdivide marketing restrictions to include bans or limits on advertising; limits on sale‐times by time of day and day of week, and on the circumstances of sale; limiting the number and type of retail sales outlets; and requiring a license to sell or serve the commodity. Attention also needs to be paid to the means of enforcement of the restrictions, for instance with a specialised agency in charge of licensing and enforcing the restrictions on sales.

## 5 Examples of Specific Measures Which Could be Explored for Wider Application

Following are some examples from Australia of legislation and other measures concerning a particular commodity which could serve as models for measures concerning other harmful commodities, or for the measure to be applied to the same commodity elsewhere.

### 5.1 Licensing and Enforcing Rules on Retail Sellers

For alcohol sales, whether served on‐premises or sold by the bottle or can for consumption elsewhere, a licensing system has long been established in Australia, as in many countries, with provision that a license can be suspended or removed for an infraction of the rules on selling—for instance, forbidding alcohol sales to children or to someone who is already drunk, or selling outside permitted sales hours. Such systems for alcohol date back to early modern times—in England, for instance, licenses to sell alcoholic beverages were first required in 1552 ([24], p. 46).

For other attractive commodities, there has often been no specific licensing scheme. The absence of a tobacco retailer licensing scheme in two Australian states, for instance, has meant that, as the cigarette taxes have been raised and applied to the legal supply, cigarettes have become a black‐market commodity, with supplies imported from abroad and sold without the tax being paid [25]. To counter this and control the market, licensing and control of retail sellers is being implemented. Thus, the state of Victoria is currently introducing a licensing scheme for those who sell cigarettes, like that for alcohol, with an agency to enforce it [26].

### 5.2 Closing off Web‐Based Sales

Commercial gambling can be sold and delivered without any tangible product to be passed between the seller and the buyer. This means that, in the age of the worldwide web, governments have little ability to control the market and sales. A substantial global web‐based gambling sales industry has thus emerged, often headquartered in island jurisdictions with few marketing controls. Australia has taken a novel approach to such offshore internet‐based commercial gambling provision. Although offshore providers are not permitted to have Australia‐based customers, these rules are ignored by many international providers [27]. To counter this, the relevant Australian national agency sends a regular notice to Australian internet service providers to block particular new or mirror sites selling gambling opportunities from diffusion in Australia, with a total of 995 such websites added to the list between November 2019 and June 2024 [28]. Although this measure has not eliminated Australian participation in cross‐border internet commercial gambling, the participation has been substantially limited.

### 5.3 Imposing a Minimum Unit Price Level

A measure which has proven value when applied to alcohol is the requirement of a minimum retail price for the product. For alcohol, this policy has primarily been applied by government levels that do not have the power to raise the excise tax on the product—for example, Scotland, Wales and the Northern Territory of Australia; governments with taxing powers prefer raising the tax, because this brings the government income. The general findings are that adopting a minimum price level does decrease alcohol consumption [29, 30]. This strategy might well be applied for other harmful products, where it would be awkward to apply a specific excise tax on the product—for instance, sugar‐sweetened beverages. Specifying a minimum unit price would be an appropriate alternative measure to mildly discourage their purchase.

## 6 Conclusion: Applying Specific Measures More Widely

The recent thinking in public health policy discussions about a wider reframing in terms of ‘unhealthy commodities’ offers the opportunity to look across what have been separate fields of action and experience in controlling markets in the interest of public health and welfare. The examples offered here, primarily from Australia, of successful measures to limit sales of harmful commodities, provide a model for developing a broader public health‐oriented literature, looking across commodities and jurisdictions, for means of limiting or reducing sale and use of harmful commodities.

There is a wide variety of specific policy measures which have been applied for one or more attractive but harmful commodity and in some countries or locales but not in others. In one jurisdiction or another, and concerning one unhealthy commodity or another, policy innovations have occurred. These measures do not necessarily have good results in terms of reducing the health and welfare burden of use of the commodity. So their effects need to be evaluated, and the results of these studies taken into account in policymaking. With such evaluation, a measure used somewhere for a particular commodity might offer a model to be adapted elsewhere and for use on another commodity.

We thus need to look more broadly at what has been done to discourage or limit the use of ‘unhealthy commodities’, because doing so can offer some new ideas for control measures. The literature on the effect and effectiveness of the measures on public health and welfare is uneven, so steps forward in applying the measures should be taken with caution, and the effects of each step evaluated. We need to establish a pattern of looking at these case studies from the perspective of ‘what is this teaching us more generally about mechanisms to reduce the rates of harmful consequences in the population?’

## Author Contributions

**Robin Room**: conceptualization, investigation, writing – original draft, writing – review and editing, data curation, methodology.

## Conflicts of Interest

The authors declare no conflicts of interest

## Data Availability

The data that support the findings of this study are available through public library.
